# The Wound Healing Effects of Iloprost in Patients with Buerger’s Disease: Claudication and Prevention of Major Amputations 

**Published:** 2011-06-01

**Authors:** A Afsharfard, M Mozaffar, F Malekpour, A Beigiboroojeni, M Rezaee

**Affiliations:** 1 Department of General and Vascular Surgery, Shohada Medical Center, Shaheed Beheshti University of Medical Sciences, Tehran, Iran; 2 Isfahan University of Medical Sciences, Isfahan, Iran

**Keywords:** Wound, Healing, Buerger’s disease, Thromboangiitis oblitrans, Iloprost

## Abstract

**Background:**

This study analyzes the therapeutic effects of intravenous infusion of iloprost in wound healing, healing of the amputation stump wound, improvement in intermittent claudication and prevention of major amputation in patients with Buerger’s disease.

**Methods:**

In a prospective study, 19 patients with known Buerger’s disease, received intravenous iloprost infusion, 6 hours per day for 10 days. Iloprost with a dose of 0.5-2 ng/kg/min according to the patients’ tolerance and using cardiac monitoring during the whole infusion period was administered for 6 hours/day using saline solution. Patients with larger wounds underwent simultaneous transmetatarsal or Ray amputation of the involved toe(s). All patients were discharged after 10 days. Patients were followed to detect their healing changes.

**Results:**

Nineteen patients, 19-55 years old received 0.5-2 ng/kg/min iloprost intravenously for 6 hours/day for 10 days. During this period, there was relative improvement in resting pain, but no significant amelioration was noticed in wound healing. In a 2 years follow-up, 14 patients showed a complete healing of the amputation stump and increased distance of walking without any pain. Some previous candidates of major amputation did not need amputation anymore. Five patients (26%) did not respond to therapy.

**Conclusion:**

Although Buerger’s disease patients who were under iloprost therapy, may not show significant changes in wound healing during treatment and at discharge, late results have proved that iloprost infusion is promising in improving wound healing and claudication and preventing major amputations.

## Introduction

Buerger’s disease or thromboangiitis oblitrans, comprises 0.5-15% of obstructive vascular diseases in western countries and 9-30% in eastern ones.[1],[2],[3] This disease is a segmental non-atherosclerotic inflammatory process which mostly involves medium and small sized arteries and veins in lower and upper extremities. It is categorized as vasculitis; patients are usually young smoker men and its prevalence in women is reported as 1-2%.[4],[5] There is a close relationship with smoking; 95% of the patients are smokers.

Its etiology is not known completely, but a strong correlation with use of every type of smoking, even non-smoked tobacco,[6],[7] has proposed the theory of sensitivity to some components of tobacco. It is not clear whether tobacco is a cause or a promoting factor, yet the active phase of the disease is related to higher level of smoking. Patients with Buerger’s disease are young to middle-aged who have a history of smoking and presents with pain while walking and in severe cases at rest, upper and lower limb discoloration and gangrene. As the possibility of vascular bypass is usually nil, the consequence of their disease is limb amputation especially in the lower extremities. Adar and colleagues showed that there is an increased cellular sensitivity to collagen types I and II in these patients when compared with normal individuals.[7] Endothelindependent vascular relaxation in peripheral vasculature is impaired in patients with Buerger’s disease.[8]

Iloprost (Ilomedin, Alprostadil) is the synthetic analogue of prostaglandin 12 (PG 12). Due to the rapid clearance from the central compartment, it is routinely administered as an intravenous infusion.[9] It is one of the hemorrheologic factors [10][11][12] which through vasodilatation [13] and prevention of platelet granule release and thromboxane A2 synthesis [14] increases the chance of delayed amputation avoidance when used in Buerger’s disease.

## Materials and Methods

Nineteen patients with the diagnosis of Buerger’s disease (from 6 months to 10 years prior to this presentation) who were all males and had a history of smoking, with presenting symptoms of burning pain of the lower extremities, intermittent claudication, toe(s) discoloration or impaired healing of the amputation stump, entered the study. Their age range was 19-55 years (mean: 38). Involved limbs were examined for the wound characteristics and its locations; all four extremities were examined and a Digital Subtraction Angiography (DSA) of the involved limb was done.

Routine lab tests and electrocardiography was checked for all patients. After explaining different treatments, the advantages and disadvantages of each and cost of the treatment with iloprost, and also after patients gave the informed consent, they were known as eligible for the study. First, the highest tolerable dose of iloprost within the range of 0.5-2 ng/kg/min was determined for each patient; this maximum dose was given as continuous intravenous infusion of iloprost in normal saline for 6 hours per day for 10 days (with tight control of vital sign and cardiac monitoring during the infusion).

An electrocardiography was done on termination of each infusion. In those patients who needed an amputation, a Ray or a transmetatarsal amputation of the toe(s) was done within the same period. The patients were discharged after 10 days. Antibiotics were given only to those patients with accompanying cellulites. Patients were followed for 2 years. The response criteria were complete wound healing, no further need for sympathectomy, improvements of claudication and significant increase in painless walking.

## Results

**Fig. 1:**
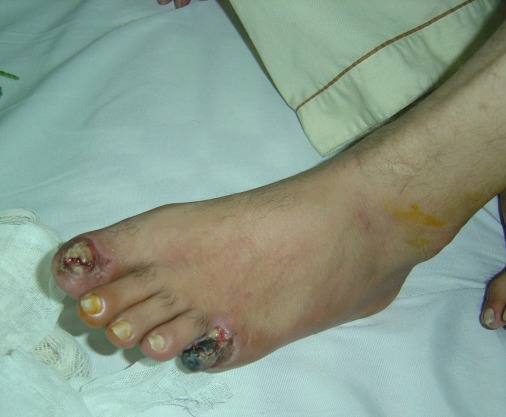
Patient with Buerger’s disease before initiation of the treatment.

Among 19 patients, one patient died 2 months after discharge from the hospital and was excluded. Patients were in the age range of 19-55 years (mean: 38) and 4 of them were above 50 years old. The rest were in their third and forth decades. They were from different locations of Iran and had a history of this illness for 6 months to 10 years. Their chief complaints varied from burning rest pain in the lower extremities, to discoloration of the limbs, intermittent claudication, and toe necrosis or amputation stump gangrene (Figure 1).

In 9 patients, popliteal pulse was not palpable in the affected limb. All patients had femoral pulses, but even in those with palpable popliteal pulse, no distal pulse could be detected. DSA was done in all patients and it was always consistent with Buerger’s vasculitis and non-reconstructable vessels. Patients had been receiving dipyridamole and pentoxiphyllin for months to years prior to this study, yet their complaints had persisted. Eleven patients had become addicted to different types of opioid analgesics due to persistent severe pain. Eight patients (42%) had a history of unior bilateral lumbar sympathectomy and one had undergone thoracic sympathectomy previously. An interval of at least four months was mandatory after sympathectomy and before bringing the patients into this study. This was supposed as the needed time for revelation of the complete effect of sympathectomy. All patients had a history of smoking between 6 months to several decades. Five patients had a past

history of below knee amputation in one of the lower extremities. In those with toe gangrene, Ray amputation of the toe was done simultaneously with the initiation of iloprost infusion. Two patients had successful skin graft placement during treatment with iloprost. After termination of iloprost infusion (10 days of treatment), patients were discharged. The signs and symptoms had some changes after 10 days: significant reduction of rest pain, decreased need to narcotic pain- killers and mild improvements of leg edema and redness. Patients felt more warmth in their legs, but there was not a dramatic change in the wound healing after 10 days. An ECG was done for each patient as a base on day 1 and every day after termination of iloprost infusion. Three patients, who developed sinus tachycardia once after infusion, responded to medications and tolerated further infusions the following days.

Liver function tests, serum biochemistry, urinalysis and coagulation tests were done for the patients on a daily basis. We encountered two cases of elevated alkaline phosphatase, one slightly and the other twice the base line. No other change in the tests was detected. In the 2 years follow-up, 5 patients did not respond to this treatment and had not improvements in wound healing. One of these cases underwent lumbar sympathectomy and transmetatarsal amputation of the first toe; while the rest 4 failures, who had undergone lumbar sympathectomy before iloprost trial, underwent below knee amputation.

**Fig. 2:**
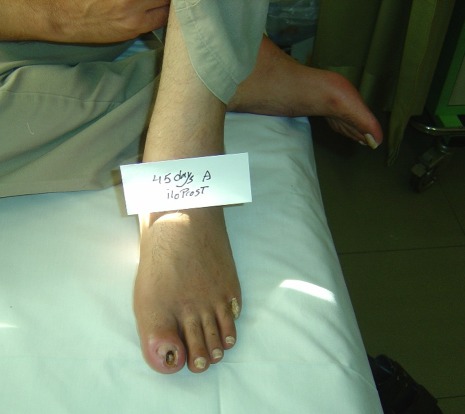
Same patient, 45 days after iloprost treatment initiation.

**Fig. 3:**
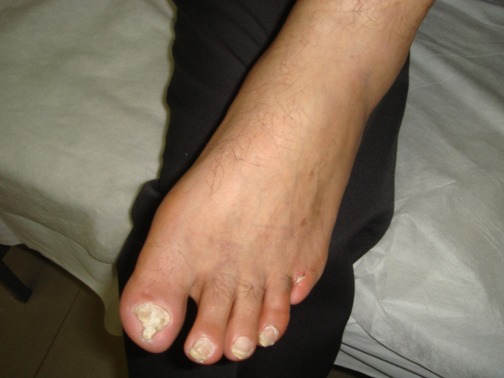
Twenty months follow-up of the patient in Figure 1.

In 13 patients (72.2%), an acceptable response to iloprost infusion was detected in form of healed wounds, improvements in intermittent claudication and pain relief in distal lower extremities (Figure 2 and Figure 3). All cases were able to successfully stop smoking.

## Discussion

Drugs effective on erythrocyte flexibility, agents acting on platelets, non-steroidal anti-inflammatory drugs (NSAIDs) and vascular reconstruction are among several therapeutic methods for Buerger’s disease. The base of treatments is ceasing smoking. All other treatments are in the second line. If the patient does not have any wound in the limb when he stops smoking, he will usually become asymptomatic.

Iloprost, a synthetic analogue of PG12, acts through vasodilatation and effects on platelets, preventing their granule release; it can improve symptoms and prevent major amputations in patients with Buerger’s disease. In this study, although the patients had not showed significant improvements in wound healing upon discharge, delayed effects of iloprost in the form of healed amputation stump wounds and avoidance of major amputations were detected within a follow-up of 2 years. [15][16]

This study was performed on 19 patients. As Buerger‘s disease is known to be more prevalent in Iran compared to western countries, we recommend that further multi-center studies be performed on larger numbers of patients.
